# Supplementary material for: Meta-Analysis and Gene Set Enrichment Relative to ER Status Reveal Elevated Activity of MYC and E2F in the “Basal” Breast Cancer Subgroup
Source: PLoS One. 2009 Mar 9;4(3):e4710. doi: 10.1371/journal.pone.0004710 (PMC2650420; doi:10.1371/journal.pone.0004710)
Supplement: Dataset S1 — GSEA results from first screen. The file contains a zipped archive of the HTML results of the second GSEA screen. Reports for enriched or depleted datasets may be accessed from the “index.html” link within the my_analysis.GseaPreranked.1219975950625.rpt.zip ZIP archive. Enrichment plots are available for each of the 50 most enriched and depleted gene sets. (13.32 MB ZIP) [file pone.0004710.s001.zip › my_analysis.GseaPreranked.1219975950625.rpt.zip!//DNA_REPLICATION_REACTOME.html]

Details for gene set DNA\_REPLICATION\_REACTOME[GSEA]

|  || Dataset | zSco.ERneg.to.ERpos\_collapsed |
| Phenotype | NoPhenotypeAvailable |
| Upregulated in class | na\_pos |
| GeneSet | DNA\_REPLICATION\_REACTOME |
| Enrichment Score (ES) | 0.5992335 |
| Normalized Enrichment Score (NES) | 2.129462 |
| Nominal p-value | 0.0 |
| FDR q-value | 7.082416E-5 |
| FWER p-Value | 0.0030 |
Table: GSEA Results Summary

  

Fig 1: Enrichment plot: DNA\_REPLICATION\_REACTOME      
 Profile of the Running ES Score & Positions of GeneSet Members on the Rank Ordered List

  

| PROBE | GENE SYMBOL | GENE\_TITLE | RANK IN GENE LIST | RANK METRIC SCORE | RUNNING ES | CORE ENRICHMENT || 1 | MCM5 |  |  | 31 | 6.533 | 0.0469 | Yes |
| 2 | ORC6L |  |  | 33 | 6.507 | 0.0958 | Yes |
| 3 | CDC45L |  |  | 108 | 5.824 | 0.1341 | Yes |
| 4 | RFC3 |  |  | 164 | 5.493 | 0.1713 | Yes |
| 5 | MCM4 |  |  | 180 | 5.436 | 0.2111 | Yes |
| 6 | MCM6 |  |  | 198 | 5.317 | 0.2498 | Yes |
| 7 | MCM10 |  |  | 201 | 5.315 | 0.2897 | Yes |
| 8 | GMNN |  |  | 206 | 5.302 | 0.3293 | Yes |
| 9 | ORC1L |  |  | 212 | 5.280 | 0.3687 | Yes |
| 10 | MCM7 |  |  | 289 | 4.970 | 0.4004 | Yes |
| 11 | RFC4 |  |  | 370 | 4.741 | 0.4301 | Yes |
| 12 | MCM2 |  |  | 630 | 4.080 | 0.4413 | Yes |
| 13 | MCM3 |  |  | 656 | 4.019 | 0.4697 | Yes |
| 14 | RFC2 |  |  | 762 | 3.821 | 0.4905 | Yes |
| 15 | CDC7 |  |  | 769 | 3.807 | 0.5187 | Yes |
| 16 | PRIM2A |  |  | 941 | 3.564 | 0.5327 | Yes |
| 17 | DIAPH2 |  |  | 1049 | 3.412 | 0.5503 | Yes |
| 18 | POLD1 |  |  | 1260 | 3.182 | 0.5585 | Yes |
| 19 | RPS27A |  |  | 1268 | 3.169 | 0.5818 | Yes |
| 20 | PCNA |  |  | 1346 | 3.087 | 0.5992 | Yes |
| 21 | RPA1 |  |  | 2380 | 2.270 | 0.5385 | No |
| 22 | CDT1 |  |  | 2522 | 2.190 | 0.5444 | No |
| 23 | ORC3L |  |  | 2573 | 2.160 | 0.5569 | No |
| 24 | POLA2 |  |  | 2649 | 2.117 | 0.5672 | No |
| 25 | CDK2 |  |  | 2688 | 2.099 | 0.5801 | No |
| 26 | CDC6 |  |  | 3185 | 1.847 | 0.5567 | No |
| 27 | ORC5L |  |  | 3377 | 1.760 | 0.5555 | No |
| 28 | POLE2 |  |  | 3975 | 1.520 | 0.5220 | No |
| 29 | RFC5 |  |  | 4034 | 1.500 | 0.5289 | No |
| 30 | ORC4L |  |  | 4538 | 1.307 | 0.5009 | No |
| 31 | RPA4 |  |  | 4610 | 1.282 | 0.5052 | No |
| 32 | RPA2 |  |  | 4690 | 1.256 | 0.5087 | No |
| 33 | POLD2 |  |  | 5531 | 0.964 | 0.4527 | No |
| 34 | RFC1 |  |  | 6197 | 0.751 | 0.4083 | No |
| 35 | PRIM1 |  |  | 6525 | 0.642 | 0.3885 | No |
| 36 | POLD3 |  |  | 6667 | 0.601 | 0.3824 | No |
| 37 | ORC2L |  |  | 6907 | 0.517 | 0.3683 | No |
| 38 | POLE |  |  | 9015 | -0.317 | 0.2119 | No |
| 39 | UBA52 |  |  | 9300 | -0.456 | 0.1940 | No |
| 40 | NACA |  |  | 10281 | -1.014 | 0.1278 | No |
| 41 | UBB |  |  | 10819 | -1.404 | 0.0979 | No |
| 42 | UBC |  |  | 10861 | -1.434 | 0.1056 | No |
| 43 | RPA3 |  |  | 11357 | -1.892 | 0.0826 | No |
| 44 | POLD4 |  |  | 13319 | -8.655 | 0.0001 | No |
Table: GSEA details [plain text format]

  

Fig 2: DNA\_REPLICATION\_REACTOME: Random ES distribution      
 Gene set null distribution of ES for **DNA\_REPLICATION\_REACTOME**
